# Preparation and Physiochemical Characterization of Bitter Orange Oil Loaded Sodium Alginate and Casein Based Edible Films

**DOI:** 10.3390/polym14183855

**Published:** 2022-09-15

**Authors:** Saurabh Bhatia, Ahmed Al-Harrasi, Mohammed Said Al-Azri, Sana Ullah, Alaa El-Din Ahmed Bekhit, Anubhav Pratap-Singh, Manish Kumar Chatli, Md. Khalid Anwer, Mohammed F. Aldawsari

**Affiliations:** 1 Natural and Medical Sciences Research Center, University of Nizwa, P.O. Box 33, Birkat Al Mauz, Nizwa 616, Oman; 2 School of Health Science, University of Petroleum and Energy Studies, Dehradun 248007, India; 3 Food Science Department, University of Otago, Dunedin 9056, New Zealand; 4 Food, Nutrition, and Health, Faculty of Land and Food Systems, 2205 East Mall, University of British Columbia, Vancouver, BC V6T 1Z4, Canada; 5 Department of Livestock Products Technology, College of Veterinary Science, Guru Angad Dev Veterinary and Animal Sciences University, Rampura Phul 151103, Punjab, India; 6 Department of Pharmaceutics, College of Pharmacy, Prince Sattam Bin Abdulaziz University, Al-kharj 11942, Saudi Arabia

**Keywords:** casein, sodium alginate, edible films, essential oil, polymers

## Abstract

Biopolymers-based composite edible films are gaining interest in the food packaging industry due to their sustainable nature and diverse biological activities. In the current study, we used sodium alginate (SA) and casein (CA) for the fabrication of composite film using the casting method. We also added orange oil to the edible film and assessed its impact on the biological, chemical, physical, and barrier properties of the films. The fabricated films were analyzed using X-ray diffraction (XRD), thermogravimetric analysis (TGA), scanning electron microscopy (SEM), and Fourier transform infrared spectroscopy (FTIR). It was observed that CA–SA films loaded with 1.5% OEO had better visual attributes, and a further increase in oil concentration was not found to be as favorable. Mechanical assessment of the films revealed that CA–SA-OEO (1.5%) film showed lower puncture deformation and higher puncture force values. XRD data showed that all samples exhibited peaks at similar positions (21° of 2θ) with different intensities. In FTIR analysis, characteristic peaks of the film components (sodium alginate, casein, and orange oil) were reported at corresponding positions. The thermal stability of films was enhanced after the addition of the OEO (1.5%), however, a greater increase in OEO caused a decrease in the thermal stability, observed during TGA analysis. Moreover, the surface of the blank CA–SA film (FL1) was found to be rough (with cracks) compared to CA–SA films (FL2) containing 1.5% OEO. Additionally, FL2 was found to be relatively better than the other samples in terms of swelling degree (SD), thickness, water solubility (WS), oxygen permeability (OP), water vapor permeability (WVP), moisture content (MC), and transparency (T).

## 1. Introduction

Recent research trends in biopolymer-based materials have shown a growing interest in food packaging [1]. Single polymer-based films often face challenges such as poor barrier and mechanical properties, whereas composite films can be designed to overcome these challenges. Composite films for food packaging are often prepared by coalescing similar or different biomacromolecules such as polysaccharides, proteins, and lipids. The majority of edible food packaging materials have been fabricated using one or multiple polysaccharides, and fewer protein–lipid, polysaccharide–protein, and polysaccharide–lipid based composite films are fabricated. Films made from protein often provide an improved oxygen barrier and mechanical properties over the films made up of polysaccharides [2,3], and that is why there is a growing interest in the protein-based composite films. However, protein based films exhibit less resistance against carbon dioxide permeability [4]. Both polysaccharides and proteins are hydrophilic macromolecules and, therefore, they have little resistance against water vapors [5]. Its physical, chemical, as well as mechanical properties are highly influenced by moisture content, which impacts the integrity and functionality of the films made of these macromolecules. Therefore, loading polysaccharides’ and proteins’ composite films with a hydrophobic material such as essential oil could be an excellent approach to improve the optical, physical, chemical, barrier, antioxidant, and antimicrobial properties. Therefore, several recent studies have reported on polysaccharides and protein based composite films that were loaded with essential oils such as ginger oil [6], cinnamon oil [7], oregano, rosemary, garlic essential oil, etc., [8] for enhancing the cohesive network and biological properties of the films [9,10]. Several approaches are used for the fabrication of edible films including two methods casting and extrusion. Moreover, for surface coating procedures such as panning, spraying, dipping, and flu-idized-bed methods are used [11,12].

In recent studies, the use of several animal proteins (gelatin, casein, and whey) and plant proteins (zein, gluten, and soy proteins) was explored for food packaging [13,14] and other biomedical applications [15], with the aim of sustainability and promotion of a greener approach [3]. Due to their nutritious nature and film forming potential, casein-based films are of great importance in the food packaging industry [16,17,18]. Films fabricated from casein are usually thin and transparent as well as possess good thermal stability, strength, and have low oxygen permeability. However, their high hydrophilicity and low elasticity affect their mechanical and barrier properties [19]. Casein-based films form a cohesive film matrix, and this matrix shrinks during the drying process and, subsequently, it becomes brittle. This cohesive matrix formed by the nonpolar and polar amino acids of the casein limits its use in food packaging applications [20].

Sodium alginate is a hydrophilic pH-responsive polymer that usually provides a strong film structure. Casein and sodium alginate are food grade edible macromolecules and can be combined to fabricate films with the best characteristics [21]. Further, the partial replacement of casein with sodium alginate will also help in lowering the cost of production of composite biodegradable films.

Due to their hydrophilic nature, a film forming solution of both polymers (casein and sodium alginate) can be easily mixed to make films with good barrier and mechanical properties [22]. However, the resultant films have a poor barrier for moisture loss. Adding essential oil to the edible films enhances the biological and barrier properties as well as improves other parameters including water solubility, swelling degree, moisture content, etc. [10]. Thus, the incorporation of optimum levels of essential oil (EO) could be an approach to impart hydrophobic character to casein–sodium alginate films. *Citrus aurantium* L., (bitter orange) oil contains high levels of D-limonene and myrcene that are known to have important biological properties, such as antimicrobial and antioxidant potential [23]. The use of orange essential (OEO) and its impact on casein–sodium alginate films has not been reported, therefore, it is important to analyze its impact and evaluate its potential use.

Similarly, the present study was designed to assess the impact of various dilutions of orange EO on the surface morphology physiochemical, mechanical, and barrier properties of casein–sodium alginate (CA–SA) composite films. The casting method was used for edible film making, and then the prepared samples were examined using various characterization techniques.

## 2. Materials and Methods

Casein (fat free ultra-purified) as well as sodium alginate (pure) were supplied by Sisco Research Laboratories Pvt Ltd., Mumbai (India). The OEO was purchased from Nature Natural India (Batch No.: NNIGIEO/104/0821). Acetic acid (98.8% pure) used in the experiment was acquired from Sigma-Aldrich, and the glycerol (99.0% pure) used in film preparation was purchased from BDH Laboratory Supplies, London (UK).

### 2.1. Preparation of Casein–Sodium Alginate–Orange Essential Oil (CA–SA–OEO) Loaded Films

The casting method was used to prepare casein (CA) and sodium alginate-based (SA) composite edible films loaded with orange oil (OEO). Initially, the CA solution (3% *w*/*v*) was made by mixing 3 g in 100 mL of distilled water (30 °C). The pH of the CA solution was adjusted to 5.6 using sodium hydroxide solution (2 N), and then it was stirred and heated at 85 °C for 10 min on a hot plate. The sodium alginate solution (3% *w*/*v*) was made by dissolving the SA in distilled water followed by heating (at 80 °C) and stirring for one hour. Both solutions (CA:SA) with a ratio of 10:1 were mixed for one hour at 80 °C, and the glycerol (2.5% *w*/*v*) was added. After this, the homogenous solution was mixed with OEO at 1.5, 2.0, and 2.5% concentration (*v*/*v*), followed by stirring (300 rpm) for one hour. The OEO loaded solution was kept at 25 °C for one hour to eliminate air bubbles [7]. After the formulation of a film-forming solution, it was cast in plastic Petri plates (90 × 15 mm) and dried at 25 °C for 48 h [4]. The films were peeled off from the surface of the Petri plates and stored in desiccators at 25 °C and 87% relative humidity. Before characterization, films were stored under the above-mentioned conditions for 12 h [9]. Table 1 shows the composition of the fabricated films. The experiment was repeated three times for the consistency of the results.

### 2.2. Thickness

Film thickness greatly impacts the thermal, mechanical, barrier, and optical properties of the films. This parameter was assessed (in mm) by a hand-operated micrometer (Mitutoyo 2046F, Kawasaki, Japan) with an accuracy of ±0.001 mm. The thickness of the film was determined at five randomly selected points before mechanical testing using the ASTM (1997) standard operating procedure [24].

### 2.3. Mechanical Properties

For determining fabricated material characteristics such as elongation at break (EAB), tensile strength (TS), as well as Young’s modulus (Ym), the texture analyzer (Stable Micro Systems Ltd., Godalming, UK) was used. The mechanical testing was conducted using ASTM standard operating procedures [25]. The samples were sliced into strips with a dimension of 40 × 10 mm and were kept at a relative humidity of 50% for a duration of 72 h. The films were held between the grips of the machine and extended at 0.75 mm/s until the break point. Distance and force values were determined. Tensile strength (TS) was evaluated using maximum force at the break point by the initial cross-sectional area (m^2^), while the elongation at the break point was measured by dividing the elongation at the break point by preliminary gauge calculation and multiplying the value by 100 [26]. The analysis was carried out in triplicate, the findings were expressed in MPa units, and the mean values were taken for statistical analysis.

### 2.4. Puncture Strength

The texture analyzer, Stable Micro Systems, Godalming, Surrey, UK (TA. XT Plus), was used to assess the puncture strength. For this measurement, a disc-shaped piece of film was placed in an annular ring clamp (3 cm diameter). The method of Lagos et al. [27] was followed for determination of deformation (mm) and puncture strength (N) of the film.

### 2.5. Water Vapor Permeability

For Water Vapor Permeability (WVP) analysis, prepared samples were sliced uniformly into a round shape having a diameter of 20 mm. Glass cups containing silica gels were sealed by the films, and then the cups were kept in the desiccator having distilled water (RH 100%) at 25 °C and weighted after a 60 min interval for 10 h. The analysis was carried out in triplicate following the ASTM (2003) guidelines. The following equation was used for the estimation of WVP:

WVP = w/t × t/(ΔP × A2)(1)

where the w/t is the weight loss per unit time (gs^−1^) measured (using linear regression where R2 was >0.99) from the water absorbed by the system until a stable state was achieved. A2 is the film area subjected to the transfer of moisture (1.539 × 10^−4^ m^2^); the film thickness is denoted by t, and ΔP represents the variation in the pressure exerted by water vapors between the two sides of the film at 25 °C (kPa).

### 2.6. Oxygen Gas Transmission Assessment

The overall resistance of the fabricated packaging material to oxygen permeability was assessed using Kurt et al.’s [28] method. This standard test is a standard sodium thiosulphate-based titration that is used to measure the oxidation of camellia oil as an indication of oxygen availability.

### 2.7. Moisture Content

Moisture content (MC) was measured from the weight variation in the samples after being dried at 105 °C. The average time duration for processing each sample was about 3–4 min. This analysis was repeated three times, and then the mean value was used for analysis.

### 2.8. Water Solubility (WS)

Water solubility (WS) of the film samples was evaluated using Singh et al.’s method [29], with some modifications. WS of the films was measured as the quantity of dry weight dissolved after 24 h of soaking in water. To determine WS, 250 mg of film sample was measured and placed in 25 mL of water for 24 h (at 25 ± 1 °C) followed by stirring (300 rpm). Afterward, the residual insoluble matter of the films was dried in an oven until a constant weight was achieved. Percent solubility (S%) was measured using the following equation:

S (%) = [(W1 − W2)/W2] × 100.(2)

W1 represents the weight of the initial dry film, whereas W2 represents the weight of undissolved films.

### 2.9. Transparency

A UV-Visible spectrophotometer (UV160U-Shimadzu, Kyoto, Japan) was used (range of 200–800 nm) for assessment of transparency. Samples with dimensions of 3 × 15 mm were placed into cuvettes and compared with a blank film (cuvette with no film). Each sample was measured in triplicate as described by Shiku et al. (2004) [30].

### 2.10. X-ray Diffraction (XRD) Studies

For analyzing the XRD pattern of fabricated edible films, a Bruker D8 Discover instrument was used. The samples were examined at a 2θ diffraction angle and 40 kV voltage and a current in the range of 5–50° at a rate of 0.500 s/point, and the Scherrer constant (K) was 1.5418 Å).

### 2.11. Microscope Observations

SEM studies were performed (JSM6510LA, SEM, Jeol, Japan) to visualize the microstructure for evaluation of features such as uniformity, compactness, pores, roughness, ridges, bulges, and particles or granules at surface or cross-section level. The analysis was carried out at an acceleration voltage of 20 kV under high vacuum mode. The samples were placed on an aluminum stub covered with adhesive tapes and gold sputtered coated.

### 2.12. FTIR Spectra Analysis

Fourier transform infrared spectra (FTIR) of the samples were carried out with an InfraRed Bruker instrument (Tensor 37, Ettlingen, Germany). The analysis was carried out at 25 ± 1 °C and the range of the wavenumber was between 400 and 4000 cm^−1^ with an average of 32 scans with a resolution of 4 cm^−1^ (data spacing 0.5 cm^−1^).

### 2.13. Thermogravimetric (TGA) Analysis

Thermal stability analysis was performed under a nitrogen atmosphere with a thermal gravimetric (TG) analyzer (TA instrument, SDT-Q600, New Castle, DE, USA). Samples were studied in a nitrogen atmosphere and scanned at a gas flow of 40 mL/min and temperature range of 25–600 °C with a ramp of 10 °C/min. A 5 mg of sample mass was used for the experiments.

### 2.14. Statistical Analysis

For all results, the mean and standard deviation (SD) values of the three independently performed experimental replicates were considered for statistical analysis. One-way ANOVA and Duncan’s test was used for the assessment of significant variations amongst mean values at 5%.

## 3. Results and Discussion

### 3.1. Visual Assessment

**Figure 1:**
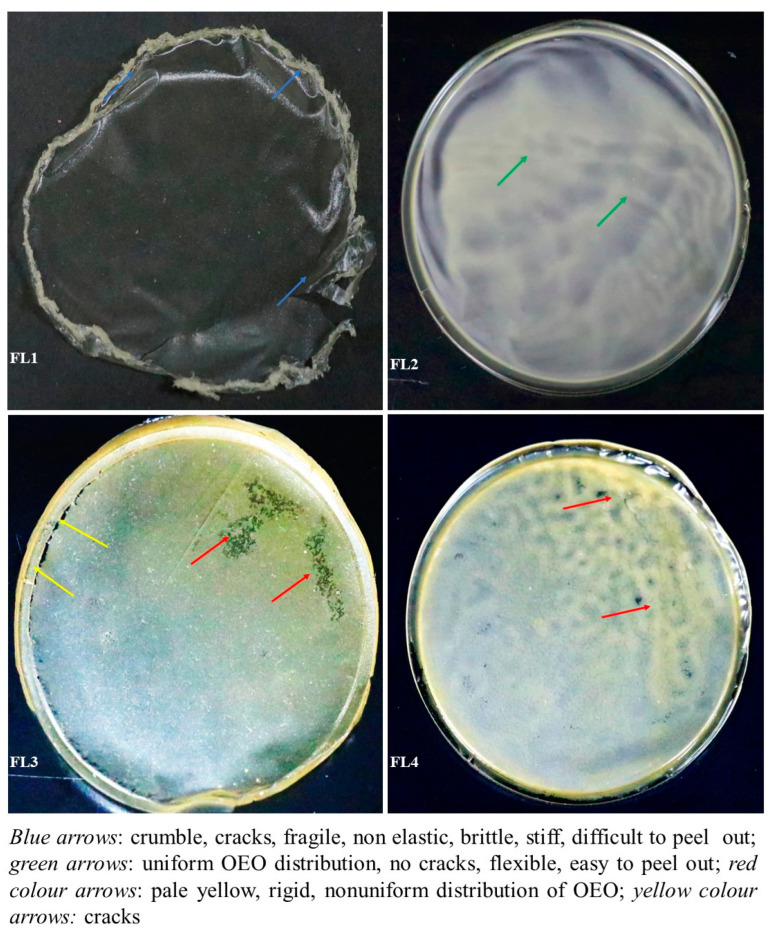
Visual assessment of films: control or FL1 (CA+SA); FL2: CA + SA + OEO (1.5% *v*/*v*); FL3: CA + SA + OEO (2% *v*/*v*); FL4: CA + SA + OEO (2.5% *v*/*v*).

The samples were visually screened, which revealed that CA–SA films loaded with 1.5% OEO had better visual attributes and were easier to peel from the petri plates. In addition, it was observed that CA–SA films with 1.5% OEO showed relatively better attributes in terms of transparency, brittleness, stiffness, flexibility, stickiness, and rigidness. Films with 2.5% OEO presented low transparency and flexibility with high stiffness, stickiness, and rigidity (Figure 1). Moreover, CA–SA film with 2.5% OEO was found to be more fragile than other films. The ratio of protein and added concentration level of OEO (1.5%) is optimum for the stability of the emulsion and the structural attributes of the film.

### 3.2. Mechanical Characteristics

Mechanical features of the food packaging material are critical to measure the structural integrity of the film and are considered as an indicator of the cohesion and durability of films. The mechanical characteristics of composite films are directed by the interaction between the molecules, which is influenced by the composition and processing methods.

Findings obtained from the elongation at break (EAB) assessment of the film containing 1.5% of OEO showed a significant (*p* < 0.05) increase, while Young’s modulus (Ym) and tensile strength (TS) decreased significantly (*p* < 0.05) (Table 2). The reduction in TS and Ym could be because of the OEO presence in the film matrix, resulting in a decrease in the inter- and intra-molecular interactions. This weakened the uniform film matrix, leading to the domination of weaker polymer–oil interactions over stronger intermolecular interactions in the film matrix [31,32] and leading to the prevention of the formation of rigid structure. Furthermore, due to the strong plasticizing effect of OEO, the plasticity was increased, which resulted in the EAB value increasing significantly (*p* < 0.05) [33]. However, films containing 2–2.5% OEO were not considerably distinct from each other in the case of mechanical properties.

### 3.3. Puncture Strength

Lower puncture distortion (PD) and higher puncture force (PF) are favorable attributes of the food packaging material that suggest high film endurance against puncture and physical handling. Films containing 1.5% OEO, relatively, showed lower puncture deformation and higher puncture force values (Table 2). However, the films containing 2–2.5% of OEO showed lesser values of films breaking force and more puncture deformation values. This suggests the concentration-dependent effect of OEO over crosslinking between the film components, as 1.5% OEO increased the crosslinking whereas higher concentration reduced intermolecular interactions, which could have made the film brittle and fragile [34]. Thus, OEO at 1.5% enhances the crosslinking between CA and SA, thereby enhancing the strength of the film, allowing for film distortion and decreasing the level of post-puncture deformation. Previously, it was observed that an increase in film thickness resulted in an increase in puncture deformation [35]. In the present study, film thickness significantly increased in FL3 to FL4 (Table 2). Thus, an increase in puncture deformation among the films (FL3 and FL4) could be ascribed to an increase in thickness [36].

### 3.4. X-ray Diffraction (XRD)

**Figure 2:**
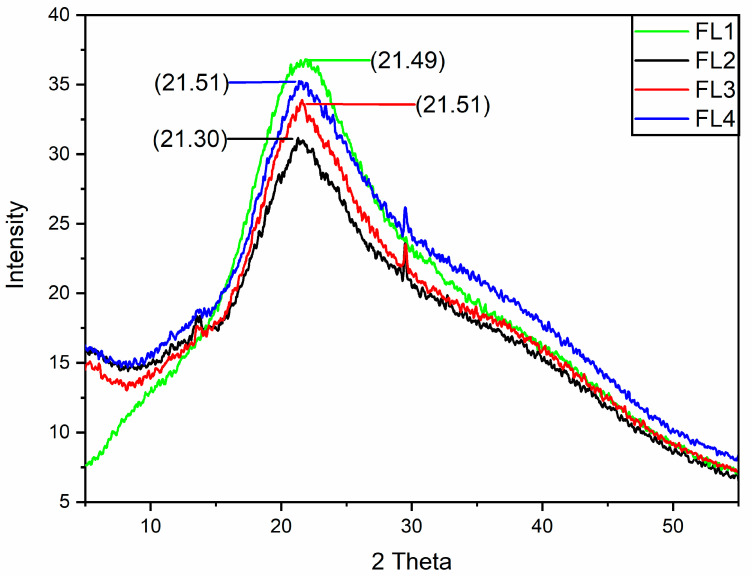
XRD analysis of FL1–FL4 samples: control or FL1 (CA + SA); FL2: CA + SA + OEO (1.5% *v*/*v*); FL3: CA + SA + OEO (2% *v*/*v*); FL4: CA + SA + OEO (2.5% *v*/*v*).

XRD offers chemical information for elemental analysis and phase analysis. For polymeric films, XRD is generally performed to determine film structure by assessing the crystal structure, degree of crystallinity, and crystallite size of the polymer [37,38]. The overlay of XRD diffraction patterns and intensities is shown in Figure 2. XRD results showed that all samples are showing peaks at similar positions with different intensities. The characteristic peaks of CA–SA and OEO loaded CA–SA based films were recorded at 21° of the 2θ position. The small characteristic peaks observed at the 14° and 29° positions could be attributed to the OEO in the respective samples. The change in peak intensity could be due to the difference in concentration of OEO added to the films. In addition, changes in mechanical characteristics of the films could also be correlated with the change in peak intensity conferred by the change in the composition of film components. Moreover, from the pattern of the peaks, it was observed that all samples had a high semi-crystalline phase. The current findings suggest that the chemical interactions among the film components are better. Previous studies have also reported a similar XRD pattern of casein–sodium alginate-based films [21,39,40].

### 3.5. FTIR Analysis

**Figure 3:**
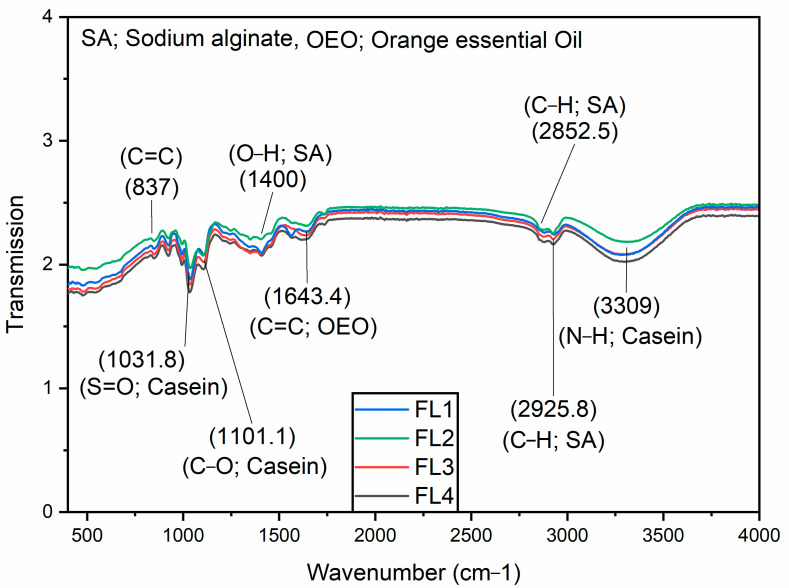
FTIR analysis of FL1–FL4 composite films samples: control or FL1 (CA + SA); FL2: CA + SA + OEO (1.5% *v*/*v*); FL3: CA + SA + OEO (2% *v*/*v*); FL4: CA + SA + OEO (2.5% *v*/*v*).

FTIR depicts the interactions between functional groups of compounds by passing an infrared wave across the material to investigate the structural changes of materials on the molecular levels. FTIR analysis revealed the characteristic peaks due to the cross-linking between sodium alginate and casein. These peaks formed owing to the N-H stretching vibration of the primary amine (3309 cm^−1^), C-H stretching of the alkane groups (2925, 2852 cm^−1^), stretching of the C=C group (1643 cm^−1^), O-H bending of carboxylic (1400 cm^−1^), strong stretching of C-O group (1101 cm^−1^), strong stretching of S=O group (1031), and C=C bending at 837 cm^−1^. Detailed FTIR information of CA–SA and OEO added CA–SA film can be seen in Figure 3. Previously, it was reported that OEO loaded films exhibited a characteristic peak at the 1644 cm^−1^ wavelength during the FTIR analysis [41]. The small shift in wavenumber (1643 to 1644 cm^−1^) could be due to the difference in concentration of the oil used. This bandwidth confirms the existence of OEO in the films, which is identified as the stretch of the C=C bond. The increase in peak intensity reflects the rise in the amount of oil. Characteristic peaks reported at the 3309 and 1031–1001 cm^−1^ positions could be attributed to the presence of casein [22,42], while peaks at 2852, 2925, and 1400 cm^−1^ positions could be attributed to the presence of sodium alginate [43]. FTIR analysis revealed the interaction of casein, sodium alginate, and OEO indicated from corresponding peak positions.

### 3.6. Thermogravimetric Analysis

**Figure 4:**
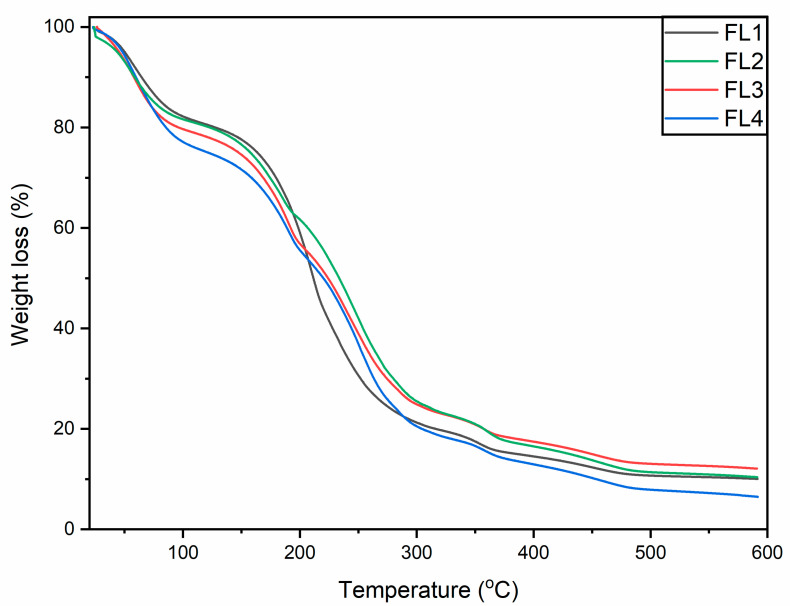
TGA thermograms of FL1–FL4 composite films samples: control or FL1 (CA + SA); FL2: CA + SA + OEO (1.5% *v*/*v*); FL3: CA + SA + OEO (2% *v*/*v*); FL4: CA + SA + OEO (2.5% *v*/*v*).

Thermogravimetric (TGA) analysis was carried out to evaluate the thermal stability of the fabricated edible films. All the samples showed similar thermal decomposition patterns, with multiple stages of thermal decomposition in the temperature range of 25–600 °C (Figure 4). Better thermal stability up to 200 °C was demonstrated by all the fabricated films. Thermal degradation was initiated at 175 °C, and the highest weight loss was seen between 200 and 250 °C. The initial phase (50–120 °C) could be attributed to the evaporation of bound water and OEO evaporation from the films. The next phase of thermal loss (120 to around 200 °C) could be associated with the glycerol loss [44]. CA–SA film decomposition could be ascribed to the subsequent stages (approximately 65% weight loss was observed), which occurred at 310–350 °C, indicating two types of chain structures with distinct thermal stabilities. The thermal stability of CA–SA film improved after the addition of the OEO (1.5%), though a greater increase in the OEO concentration caused a decline in thermal stability.

### 3.7. Microstructure of CA–SA and OEO–CA–SA Films

**Figure 5:**
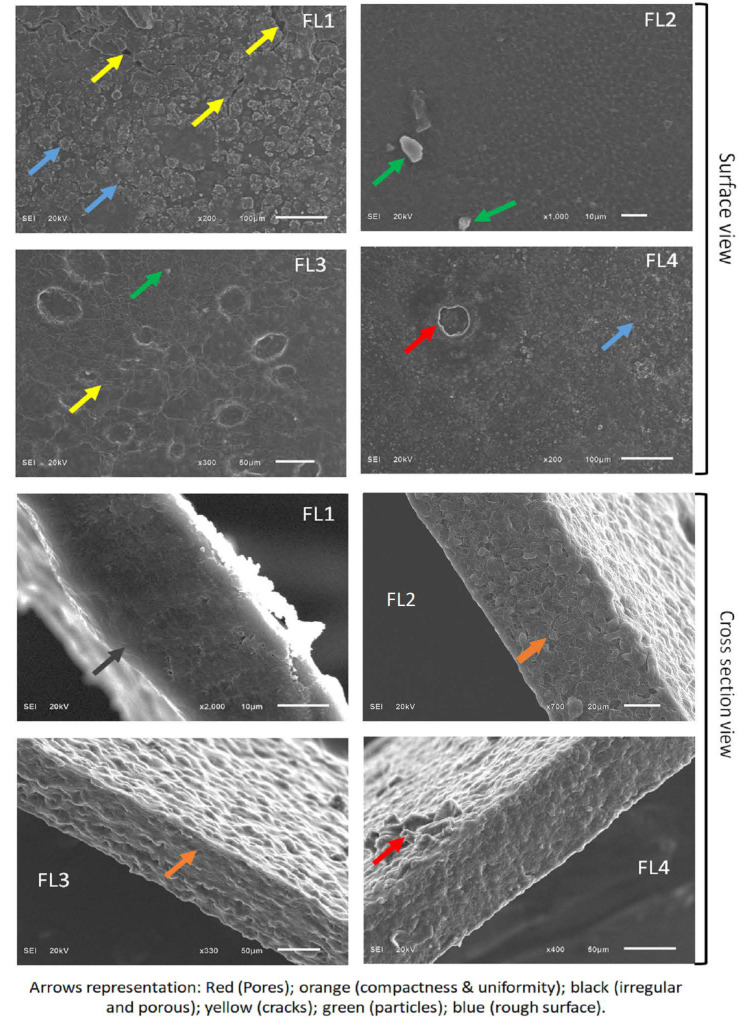
SEM analysis of FL1–FL4 composite films samples: control or FL1 (CA + SA); FL2: CA + SA + OEO (1.5% *v*/*v*); FL3: CA + SA + OEO (2% *v*/*v*); FL4: CA + SA + OEO (2.5% *v*/*v*).

Surface morphology is based on the chemical interaction between film components, which considerably impacts the overall properties of the film [45]. Thus, to establish a relationship between morphological features and the performance of the film, an SEM investigation was conducted. Surface as well as cross-section images of CA–SA and OEO–CA–SA films are illustrated in Figure 5. The surface of the CA–SA films (FL1) was rough and uneven with cracks, whereas CA–SA films (FL2) containing 1.5% OEO have smooth surfaces with few particles. However, CA–SA films containing 2–2.5% OEO (FL2, FL3) present large size holes, possibly due to the volatilization of free OEO. Additionally, the films containing 2–2.5% OEO revealed a rough surface with minor fractures and wrinkles as well as big holes. These defects at the films’ surface could be ascribed to the emulsification, flocculation, and coalescence of OEO in the fabricated material during drying, leading to droplet formation and volatilization of OEO [46]. On other hand, CA–SA films containing 1.5% OEO (FL2) showed uniform and compact structure with a smooth surface and without any cracks and pores. It was assumed that OEO addition (1.5%) caused a modification in the structural organization of the film, which could be associated with changes in WVP and TS values.

### 3.8. Film Thickness

Film thickness increased 42.01–81.87 μm with the rise in the concentration of OEO content (*p* < 0.05). This could be due to the entrapped micro-droplets formation of OEO in the polymeric matrix and due to the interaction between the film components and OEO [47]. This finding is in line with the study based on the increase in thickness of films after the supplementation of thyme essential oil to starch film [48].

### 3.9. Swelling Degree

Swelling is an unwanted attribute for a biopolymer-based film, mainly if meant for packaging food with high MC [49]. In the present work, a significant increase in SD values has been observed in films containing EO from 2% to 2.5%. However, films containing 1.5% OEO have not shown an increase in SD values. This might be associated with the surge in the availability of free OEO in films containing OEO of 2 to 2.5%. This unbound EO might have possibly reduced cross-linking between CA–SA and, thus, decreased cohesion forces of the polymeric chain causing the formation of a heterogeneous matrix with an increase in SD values. However, unlike the effect of OEO (2–2.5%) over CA–SA films, 1.5% of OEO film has not shown an increase in SD value. This behavior of OEO film at 1.5% could be due to the boost in intermolecular interaction due to the participation of OEO components in bridging the strong interaction between CA–SA.

### 3.10. Water Vapor Permeability and Water Solubility

The solubility of the films and their resistance against water vapors impact the physical and chemical characteristics of the films specifically in an environment with high humidity. In the present study, it was found that solubility declined significantly (*p* < 0.05) with increasing OEO (Table 3). These findings suggested that the possible addition of OEO improved the water resistance of the films. It could be due to the increase in hydrophobicity of the films caused by the supplementation of the OEO. Additionally, chemical interaction between hydroxyl groups of CA–SA and components of OEO decreased the interaction of hydroxyl groups with water molecules, resulting in low solubility of the films [50].

WVP is a crucial parameter of food packaging films that impacts product safety as well as quality via moisture retention or loss. Water transmission usually arises through the hydrophilic portion of the packaging material and is, therefore, dependent on the hydrophilic/hydrophobic ratio of the film. As demonstrated in Table 3, since OEO is hydrophobic, increasing the OEO level in the films resulted in a significant decrease in WVP of films containing 1.5% of EO. This behavior could be due to the higher tortuosity made within the CA–SA polymeric matrix as the water vapor molecules passes, resulting in a reduction in permeation [51].

Further rise in WV transmittance in the films containing 2.0–2.5% of OEO could be because of the adverse impact of OEO on the micro-structural features of fabricating material and the creation of micropores (confirmed from SEM images, Figure 5) as well as pores in the film structure, leading to movement of WV molecules [52]. An increase in water vapor transmittance could be also attributed to discontinuation caused in the polymeric matrix by the OEO droplets, resulting in the drop in the solidity and enhancement of the transmission process of the films containing 2.5% OEO [53].

### 3.11. Oxygen Barrier Property

The oxygen barrier property (OBP) of edible films should be the minimum required to protect the food from unfavorable oxidation reactions [54]. Casein-based films often possess low OBP, however, its blend with SA and OEO has not been investigated yet [20]. As per a previous report, structural changes in casein and type of plasticizer have a significant impact on OP [55]. It was also found that lactic acid–casein EFs plasticized with sorbitol possessed more effective oxygen barrier properties [56]. However, another report suggested lower oxygen permeability of casein films than casein–whey protein composite films, irrespective of the plasticizer used [57]. In the present study, initially, CA–SA-OEO (1.5%) possessed lower OP than films containing 2–2.5% of OEO. An increase in OP could be because of using OEO as a plasticizer and swelling of films because of the volatilization of OEO. Oxygen may pass via CA–SA interfaces, offering oxygen-penetration channels when higher concertation of OEO is added to the films [58].

### 3.12. Moisture Content

Casein based films are extremely sensitive to moisture content. They readily absorb and release water molecules, which act as a plasticizer and significantly impact the characteristics of the films [20]. In the present work, OEO loaded CA–SA films presented less moisture content than blank films (Table 3). This behavior could be due to the formation of covalent bonding between the CA–SA chains and OEO, resulting in the reduction in the accessibility of amine and hydroxyl groups in the film matrices. The covalent bonding between OEO and the polymeric matrix decreased the hydrogen bonding between water molecules and the functional groups of polymeric chains, ultimately leading to a decrease in the moisture content of films loaded with OEO [59].

### 3.13. Transparency

Transparency is an important attribute of the films meant for food packaging material, as this parameter impacts consumer acceptability and the transmission of radiations from packaging material to food. Light transmittance of FL1–FL4 was shown in Table 3. FL1 without EO showed 56.38% light transmittance, which was the highest among all samples. An increase in the concertation of OEO from 1.5 to 2.5% (*w*/*v*) significantly decreased the films’ transmittance (*p* < 0.05). The decrease in transmission of light could be attributed to the scattering of light at the interface of OEO droplets that were present in the polymeric matrix of the film [52]. Moreover, color components present in OEO perhaps reduced the transparency of the films. These findings are in line with findings reported by Pirouzifard et al. [60]. These findings demonstrated that the OEO could effectively prevent the transmission of UV rays across the film, and thus this packaging material can effectively prevent food oxidation caused by UV rays, especially in light-sensitive food.

## 4. Conclusions

In the current study, we fabricated composite films containing casein and sodium alginate that were supplemented with orange essential oil. We characterized the films using different physiochemical techniques. It was concluded that the casein–sodium alginate based edible film (FL2) containing 1.5% orange oil was comparatively better than the other samples in terms of morphological characteristics, thermal stability, and mechanical properties. In addition, FL2 also revealed better barrier properties, transparency, and water vapor permeability compared to other films. To promote sustainability, the OEO loaded biopolymer-based films could be used for food packaging applications. The current work is limited to only the physiochemical characterization, mechanical, and barrier properties of the fabricated films and lacks information related to the antibacterial and antioxidant potential of OEO.

## Figures and Tables

**Table 1:** Composition of the OEO-loaded CA–SA-based composite films.

Codes	Film Composition
FL1	CA+SA
FL2	CA + SA + OEO (1.5% *v*/*v*)
FL3	CA + SA + OEO (2% *v*/*v*)
FL4	CA + SA + OEO (2.5% *v*/*v*)

CA: casein; SA: sodium alginate; OEO: orange essential oil.

**Table 2:** Mechanical properties of CA–SA and CA–SA–OEO films.

Sample Codes	EAB (%)	TS (MPa)	Ym	PF (N)	PD (%)
FL1	7.21 ± 0.12 ^c^	8.21 ± 0.02 ^a^	98.34 ± 3.14 ^a^	4.87 ± 0.07 ^b^	78.21 ± 3.78 ^a^
FL2	19.22 ± 1.37 ^b^	6.22 ± 0.01 ^b^	45.13 ± 1.77 ^b^	6.71 ± 0.01 ^a^	23.89 ± 1.31 ^c^
FL3	20.11 ± 0.78 ^a^	5.42 ± 0.04 ^c^	43.17 ± 2.36 ^b^	4.82 ± 0.02 ^b^	54.32 ± 0.67 ^b^
FL4	20.32 ± 0.63 ^a^	4.91 ± 0.03 ^d^	42.21 ± 4.15 ^b^	3.77 ± 0.18 ^c^	75.62 ± 2.24 ^a^

Composite films samples: control or FL1 (CA + SA); FL2: CA + SA + OEO (1.5% *v*/*v*); FL3: CA + SA + OEO (2% *v*/*v*); FL4: CA + SA + OEO (2.5% *v*/*v*). EB: elongation at break, Ym: Young’s modulus; TS: tensile strength, PD: puncture deformation; PF: puncture force. Findings are stated as means ± standard deviation. The letters a–d (superscript) represents the difference between the mean values (*p* < 0.05).

**Table 3:** Thickness, water vapor permeability (WVP), oxygen barrier property (OBP), water solubility (WS), swelling degree (SD), moisture content (MC), and transparency (T) of CA–SA and CA–SA–OEO films.

Sample Codes	Thickness (μm)	SD (%)	WS (%)	WVP (×10^−12^ g·cm/cm^2^·s·Pa)	OP (g/100 g)	MC (%)	T
FL1	42.01 ± 2.4 ^c^	77.1 ± 3.71 ^d^	74 ± 2.14 ^a^	2.71 ± 0.01 ^a^	1.89 ± 0.1 ^c^	16.12 ± 0.61 ^a^	56.38 ± 2.38 ^a^
FL2	46.32 ± 1.7 ^c^	81.2 ± 2.78 ^c^	32 ± 1.56 ^b^	1.27 ± 0.03 ^b^	1.45 ± 0.3 ^c^	10.21 ± 0.34 ^b^	31.23 ± 1.02 ^b^
FL3	63.91 ± 2.6 ^b^	132.4 ± 1.86 ^b^	28 ± 2.33 ^c^	1.58 ± 0.01 ^b^	2.54 ± 0.1 ^b^	9.63 ± 0.72 ^c^	21.08 ± 1.33 ^c^
FL4	81.87 ± 3.1 ^a^	139.1 ± 3.41 ^a^	29 ± 1.42 ^c^	2.25 ± 0.04 ^a^	3.81 ± 0.2 ^a^	9.55 ± 0.24 ^c^	18.11 ± 1.89 ^d^

Composite films samples: control or FL1 (CA + SA); FL2: CA + SA + OEO (1.5% *v*/*v*); FL3: CA + SA + OEO (2% *v*/*v*); FL4: CA + SA + OEO (2.5% *v*/*v*). Findings are stated as means ± standard deviation. The letters a–d (superscript) represents the difference between the mean values (*p* < 0.05).

## Data Availability

The corresponding author could be approached for the availability of data.
